# Mutual Expression of ALDH1A1, LOX, and Collagens in Ovarian Cancer Cell Lines as Combined CSCs- and ECM-Related Models of Drug Resistance Development

**DOI:** 10.3390/ijms20010054

**Published:** 2018-12-23

**Authors:** Karolina Sterzyńska, Andrzej Klejewski, Karolina Wojtowicz, Monika Świerczewska, Marta Nowacka, Dominika Kaźmierczak, Małgorzata Andrzejewska, Damian Rusek, Maciej Brązert, Jacek Brązert, Michał Nowicki, Radosław Januchowski

**Affiliations:** 1 Department of Histology and Embryology, Poznan University of Medical Sciences, Święcickiego 6 St., 61-781 Poznań, Poland; kwojtowicz@ump.edu.pl (K.W.); mswierczewska@ump.edu.pl (M.Ś.); martarozmiarek@poczta.onet.pl (M.N.); dominika.ka.poznan@gmail.com (D.K.); mandrzej@ump.edu.pl (M.A.); mnowicki@ump.edu.pl (M.N.); rjanuchowski@ump.edu.pl (R.J.); 2 Department of Nursing, Poznan University of Medical Sciences, Smoluchowskiego 11 St., 60-179 Poznań, Poland; aklejewski@ump.edu.pl; 3 Department of Obstetrics and Women’s Diseases, Poznan University of Medical Sciences, Polna 33 St, 60-535 Poznań, Poland; 4 Department of Pathomorphology, Regional Hospital in Poznań, Juraszów 7 St, 60-479 Poznań, Poland; d.rusek@vp.pl; 5 Division of Infertility and Reproductive Endocrinology, Department of Gynecology, Obstetrics and Gynecological Oncology, Poznan University of Medical Sciences, Polna 33 St, 60-535 Poznań, Poland; maciejbrazert@ump.edu.pl; 6 Department of Gynecology, Poznan University of Medical Sciences, Polna 33 St, 60-535 Poznań, Poland; jbrazert@ump.edu.pl

**Keywords:** lysyl oxidase (LOX), aldehyde dehydrogenase (ALDH1A1), collagen, cancer stem cells (CSCs), extracellular matrix (ECM), ovarian cancer

## Abstract

A major contributor leading to treatment failure of ovarian cancer patients is the drug resistance of cancer cell. CSCs- (cancer stem cells) and ECM (extracellular matrix)-related models of drug resistance are described as independently occurring in cancer cells. Lysyl oxidase (LOX) is another extracellular protein involved in collagen cross-linking and remodeling of extracellular matrix and has been correlated with tumor progression. The expression of LOX, COL1A2, COL3A1, and ALDH1A1 was performed in sensitive (A2780, W1) and resistant to paclitaxel (PAC) (A2780PR1 and W1PR2) and topotecan (TOP) (W1TR) cell lines at the mRNA (real-time PCR analysis) and protein level (Western blot and immunofluorescence analysis). The ALDH1A1 activity was measured with the ALDEFLUOR test and flow cytometry analysis. The protein expression in ovarian cancer tissues was determined by immunohistochemistry. We observed an increased expression of LOX and collagens in PAC and TOP resistant cell lines. Subpopulations of ALDH1A1 positive and negative cells were also noted for examined cell lines. Additionally, the coexpression of LOX with ALDH1A1 and COL1A2 with ALDH1A1 was observed. The expression of LOX, collagens, and ALDH1A1 was also detected in ovarian cancer lesions. In our study LOX, ALDH1A1 and collagens were found to be coordinately expressed by cells resistant to PAC (LOX, ALDH1A1, and COL1A2) or to TOP (LOX and ALDH1A1). This represents the study where molecules related with CSCs (ALDH1A1) and ECM (LOX, collagens) models of drug resistance are described as occurring simultaneously in ovarian cancer cells treated with PAC and TOP.

## 1. Introduction

Ovarian cancer is the most lethal gynecological malignancy and the leading cause of the gynecological cancer-related death among women [1]. The high mortality rate results from late diagnosis at the advanced clinical stage of the disease with peritoneal spread or distant metastases [2,3]. Regardless of the clinical stage the standard treatment includes the surgical cytoreduction followed by chemotherapy based on platinum (Cisplatin-CIS or carboplatin) and taxol (Paclitaxel—PAC) compounds [4]. Unfortunately, despite the initial response to first-line therapy most tumors often develop resistance to these drugs. In case of platinum resistance, the second line chemotherapy includes other drugs like topotecan (TOP) and doxorubicin (DOX) [5,6]. Unfortunately, a response to second line of chemotherapy is usually low and accounts from 15 to 35%.

Drugs used in the first and the second line of chemotherapy show different mechanisms of action. CIS causes the intra- and interstrand DNA cross-linking that results in blocking DNA replication and RNA transcription [7]. PAC binds to β-tubulin subunits leading to microtubule stabilization and blocking the mitosis [8]. TOP and DOX stabilize DNA-topoisomerase complexes by formation of irreversible covalent cross-links leading to inhibition of DNA replication [9]. Eventually, all these agents should cause the apoptotic death of cancer cells. Unfortunately, cancers develop a plethora of drug resistance mechanisms that are related to different level of tumor organization and can be in general divided into the cancer cell specific and cancer tissue specific ones. The cellular mechanisms of action are to decrease a drug concentration in the cell, inactivate the drug or to repair the damages made by drug. The most significant way of lowering the drug concentration is to actively remove the drug from cancer cells by drug transporters from ABC family [10]. Among the most important are glycoprotein P (P-gp) responsible for removal of PAC, DOX [11,12], and probably TOP [13], breast cancer resistant protein (BCRP) that removes TOP [14], as well as MRP2 playing role in CIS resistance [15]. The expression of above-mentioned transporters was also observed by ours in drug-resistant ovarian cancer cell lines [12,13,16]. The tumor shows heterogeneous structure where the anatomical and histological organization can create a set of barriers that block drug delivery. The solid stress induced by growing tumor, dense cellular structure and changes in the microenvironment can limit drug delivery to cells localized in the center of the tumor. [17]. Since the tumor is characterized by abundant expression of extracellular matrix components (ECM) (e.g., proteoglycans, lamins, and collagens) the drug diffusion and speed delivery is diminished [18,19]. Furthermore, some cytotoxic drugs (DOX and PAC) can be directly bound by ECM components that limit their availability to the cancer cells [20]. The interaction of cancer cells with ECM activates the intracellular signaling leading to cell adhesion-mediated drug resistance (CAM-DR) [21,22]. The expression of ECM components is not limited only to cells found within the cancer microenvironment but was also observed to be expressed by cancer cells [23,24,25,26,27].

Among the many ECM proteins discovered lately as being important in tumorigenesis lysyl oxidase (LOX) was found. LOX is a copper-dependent amine oxidase that belongs to the LOX family composed of five proteins (LOX, LOXL, LOXL2, LOXL3, and LOXL4) [28] where LOX was the most studied in regard its physiological and pathological role. The protein is synthesized as the 48kDa preproenzyme (preproLOX), N-glycosylated, and secreted outside the cell as the inactive 50kDa proenzyme (proLOX). The extracellular proLOX has no catalytic activity and requires activation by proteolytic cleavage by procollagen C-proteinase. In this way, the mature 32 kDa protein (LOX) and the 18 kDa propeptide (LOX-PP) are formed [29,30]. The mature 32 kDa enzyme is responsible for initiation of covalent cross-linking of collagens and elastin in the extracellular matrix that results in increase in tensile strength and ECM stabilization [30]. It has been shown that LOX is involved not only in extracellular but also in intracellular activities, and as the secreted protein can re-enter the cell and influence the cell signaling and gene expression [30,31,32]. Regarding the literature data, the role of LOX in cancer seems to be dual, since both down- and upregulation of LOX in cancer cell lines and tumor tissues has been described. The potential role of LOX as the tumor suppressor has been described for example in colon [33], pancreatic [34], basal and squamous cell carcinomas [35]. On the other hand, the high expression of LOX was described as the metastasis promoter in lung adenocarcinoma [36], breast [37,38], colorectal [39], and ovarian cancer [40,41]. In all these studies, higher expression of LOX has been correlated with tumor grade, invasion and migration of cancer cells, increased metastatic disease, and decreased survival.

Currently at least three independent models of drug resistance development in cancer are described in the literature. The classic one establishes that all the cells in the tumor have the same chance to develop drug resistance after drug exposure [42]. According the second model—the CSCs (cancer stem cells) model—only a small population of cells is resistant to chemotherapy before treatment and the content of these cells increases after therapy [43,44]. The characteristic feature of CSCs is very high level of drug transporters from the ABC family, especially P-gp and BCRP [45]. As the most universal marker of CSCs in solid tumors the high expression of ALDH1A1 and/or ALDH3A1 enzymes is considered [43,46]. Many research indicates the role of ALDH1A1 in the cancer biology where increased expression is related to drug resistance in many solid tumors and negatively correlates with progression and overall survival [47,48]. The third model of drug resistance describes the role of ECM compounds produced by cancer cells [22]. The cells demonstrating high expression of ECM preferentially survive cytotoxic treatment because of increased resistance. Furthermore, they can induce drug resistance in surrounding cells by CAM-DR [22].

Previously we have described a TOP-resistant ovarian cancer cell line showing high expression of the drug transporter BCRP along with the subpopulation of ALDH1A1-positive cells [49]. From the other hand, all the cells expressed COL3A1, but the most abundant expression was observed also in ALDH1A1 positive cells [27]. In this study, we described expression of another ECM molecule—LOX—in PAC- and TOP-resistant ovarian cancer cell lines. In the cell line resistant to PAC (A2780PR1) a clear correlation between LOX, ALDH1A1, and COL1A2 expression was observed. In another examined cell line resistant to PAC (W1PR2) and TOP (W1TR) increased expression of LOX was also observed among ALDH1A1 positive cells. Hereby, we combined both models of drug resistance development in the ovarian cancer.

## 2. Results

### 2.1. LOX Gene and Protein Expression in Drug-Resistant Ovarian Cancer Cell Lines

**Figure 1:**
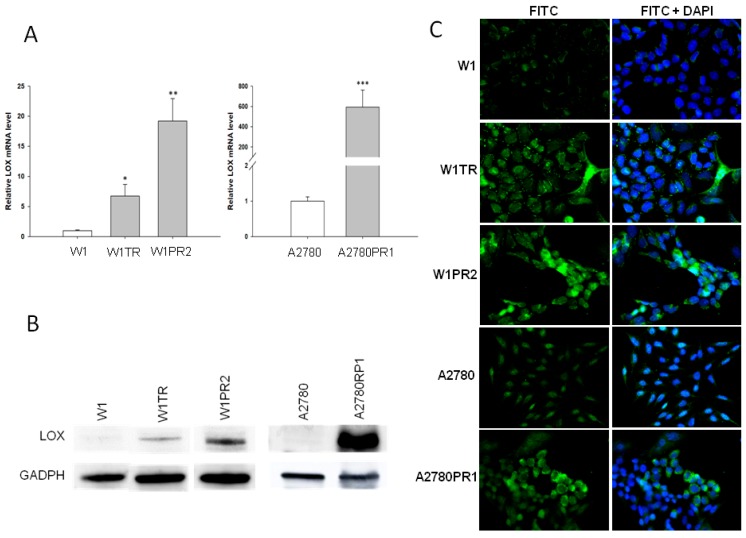
Expression analysis of (**A**) *LOX* transcript (Q-PCR) in the W1, A2780, and drug-resistant cell sublines. The figure presents the relative gene expression in the resistant cell lines (gray bars) with respect to that in the sensitive cell line (white bars), which has been assigned a value of 1. The values were considered significant at * *p <* 0.05, ** *p <* 0.01, and *** *p* < 0.001. (**B**) LOX protein expression analysis in the W1, A2780, and drug-resistant cell lines. The cellular proteins were separated using 7% PAGE and transferred to a PVDF membrane, which was then immunoblotted with either primary Ab or HRP-conjugated secondary Ab. A primary anti-GADPH Ab was used as a loading control for the cell lysates. (**C**) LOX immunofluorescence in the W1 and A2780 drug-resistant cell sublines. LOX was detected using the anti-LOX antibody and Alexa Fluor^®^488-conjugated secondary antibody (green). To visualize the cell nuclei, the cells were mounted with a DAPI-containing mounting medium (blue). Objective 40×.

To determine whether the development of drug resistance in W1 and A2780 drug-resistant sublines is associated with *LOX* overexpression, the expression of the *LOX* mRNA was assessed. We observed a statistically significant increase of the *LOX* transcript in W1 TOP- and PAC-resistant cell lines (*p* < 0.05 and *p* < 0.01, respectively) and in A2780 PAC-resistant cell line (*p* < 0.001; Figure 1A). However, the expression of *LOX* was variable in these cell lines. We observed approximately seven- and nineteen-fold higher transcript levels in the W1TR and W1PR2 cells, respectively, when compared to the control. Expression in the A2780PR1 cells increased about 600-fold in comparison to the A2780 cell line. The elevated expression of LOX at the protein level was confirmed by western blot analysis. We observed some increase in LOX bands intensity in both PAC- and TOP-resistant W1 cell lines. A considerable increase in LOX band intensity was observed in the A2780PR1 cell line (Figure 1B). However, detection of LOX in the W1PR2 and W1TR cell lines required longer exposure than in A2780PR1 cell line. In all resistant cell lines, we observed correlation between transcript and protein level. The Western blot results are informative for the expression of the investigated protein among the whole cell population; however, the result may not correspond with the expression of particular proteins among the whole cell population. To determine the expression of the LOX protein in the investigated cell lines, we performed fluorescence analysis in W1, W1TR, and W1PR2 as well as in A2780 and A2780PR1 cell lines. The low, almost detectable, fluorescence signal was present in the W1 and A2780 cell lines (Figure 1C). In the W1TR, W1PR2, and A2780PR1 cell lines, we observed an increase in fluorescence intensity. However, in all three resistant cell lines two cell subpopulations differing in fluorescence intensity were noticed. In W1TR, W1PR2, and A2780PR1 cell lines the uniform increased expression was observed for majority of cells together with individual cells presenting very strong fluorescent signal (Figure 1C).

### 2.2. Early Response to Cytotoxic Drug Treatment in Ovarian Cancer Cell Line

**Figure 2:**
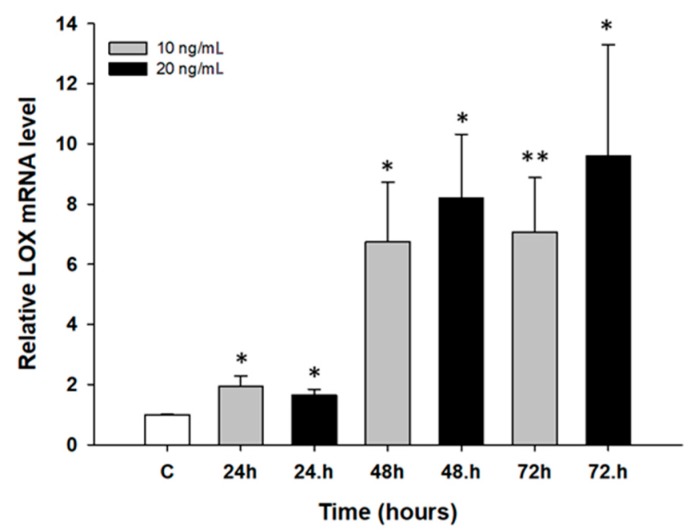
Expression analysis of the *LOX* gene in the W1 cell line after short time exposure to PAC. The figure presents relative genes expression in PAC treated cells (gray and black bars) with respect to the untreated control (white bars) assigned as 1. The values were considered significant at * *p <* 0.05 and ** *p* < 0.01.

The next step was to determine the early response of drug-sensitive cell lines to PAC and TOP treatment. In time course experiments, W1 and A2780 cell lines were treated with low concentrations of PAC (20 ng/mL and 25 ng/mL) and of TOP (10 ng/mL and 20 ng/mL) for 24, 48, and 72 h. Afterwards, *LOX* gene expression analysis was performed. We did not observe any significant changes in gene expression in dose dependent manner after TOP treatment in both cell lines and PAC treatment in A2780 cell line. However, we observed a time-dependent increase in *LOX* transcript after short time exposure to PAC in W1 cell line (*p* < 0.05 or *p* < 0.01; Figure 2). 

### 2.3. ALDH1A1 Gene and Protein Expression in Drug-Resistant Ovarian Cancer Cell Lines

**Figure 3:**
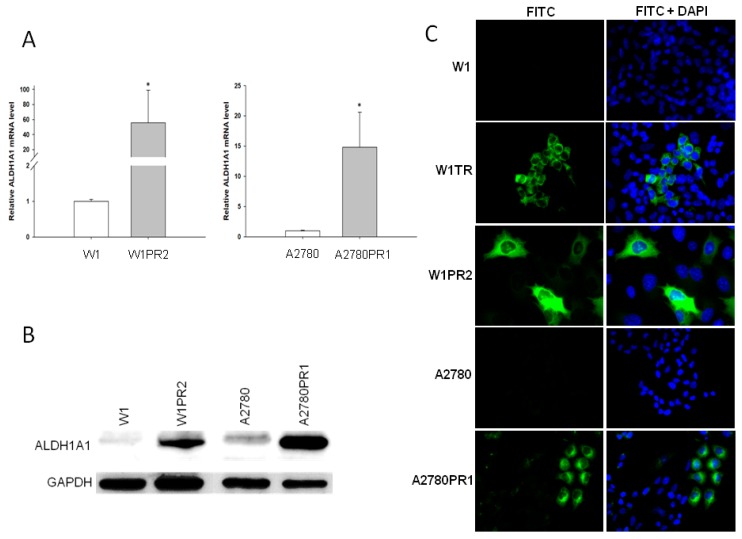
Expression analysis of (**A**) *ALDH1A1* transcript (Q-PCR) in the W1, A2780, and their sublines resistant to PAC. The figure presents the relative gene expression in the resistant cell lines (gray bars) with respect to that in the sensitive cell line (white bars), which has been assigned a value of 1. The values were considered significant at * *p <* 0.05. (**B**) ALDH1A1 protein expression analysis in the W1, A2780, and their sublines resistant to PAC. The cellular proteins were separated using 7% PAGE and transferred to a PVDF membrane, which was then immunoblotted with either primary Ab or HRP-conjugated secondary Ab. A primary anti-GADPH Ab was used as a loading control for the cell lysates. (**C**) ALDH1A1 immunofluorescence in the W1 and A2780 drug-resistant cell sublines. ALDH1A1 was detected using the anti-ALDH1A1 antibody and Alexa Fluor^®^488-conjugated secondary antibody (green). To visualize the cell nuclei, the cells were mounted with a DAPI-containing mounting medium (blue). Objective 40×.

Previously we observed increased expression of the *ALDH1A1* gene, which is a marker of CSCs in the W1TR cell line [49]. Therefore, in the present study we have investigated *ALDH1A1* gene expression in another two drug-resistant cell lines: W1PR2 and A2780PR1. To determine whether the development of drug resistance in W1 and A2780 drug-resistant sublines is associated with *ALDH1A1* gene overexpression; expression of the *ALDH1A1* mRNA was assessed. We observed a statistically significant increase of the *ALDH1A1* transcript in W1 PAC-resistant cell line (*p* < 0.05) and in A2780 PAC-resistant cell line (*p* < 0.05) (Figure 3A). Approximately 55- and 15-fold higher transcript levels were noticed in the W1PR2 and A2780PR1 cells, respectively, as compared to the control cell lines. In contrast, about 1000-fold increase was observed in W1TR cell line previously [49]. The elevated expression of ALDH1A1 was confirmed at the protein level by Western blot analysis We could observe a considerable increase in ALDH1A1 band intensity in both PAC-resistant cell lines when compared to drug-sensitive cell lines (Figure 3B). The ALDH1A1 protein expression was also confirmed at the cellular level in the immunofluorescence experiment. We did not detect any fluorescence signal in drug-sensitive W1 and A2780 cell lines (Figure 3C). In the drug-resistant cell lines the expression of ALDH1A1 protein was increased; however, different distribution of fluorescence intensity was noticed dependently on the cell line. In W1TR cell line two subpopulations of cells, one with high ALDH1A1 expression and another with no ALDH1A1 expression, were observed. The W1PR2 cell line showed three cell subpopulations with regard to fluorescence intensity. The groups of cells with enormous expression of ALDH1A1, other with medium ALDH1A1 fluorescence intensity, and another without ALDH1A1 expression, were observed. Similar cells populations were also observed in A2780PR1 cell line (Figure 3C).

### 2.4. Flow Cytometry Analysis of the ALDH1A1 Population

**Figure 4:**
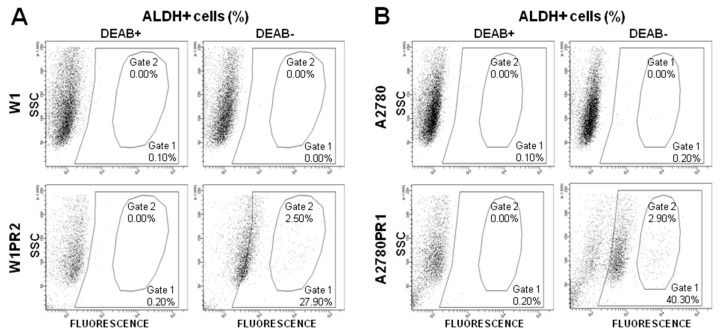
Flow cytometry analysis of ALDH+ cell content in W1 and W1PR2 (**A**) as well as in A2780 and A2780PR1 cell lines (**B**) DEAB, a specific inhibitor of ALDH, was used for confirmation of gating areas. Fluorescence was measured using a fluorescein isothiocyanate (FITC) channel.

To determine the number of cells with low, medium, and high enzymatic activity of ALDH1A1 in the investigated cell lines, we subjected cells to flow cytometric analysis using the ALDEFLUOR assay, which identifies active ALDH1A1. Two different ALDH1A1 populations we observed in W1TR cell line [49]. In this study, in the W1PR2 cell line we observed a subpopulation of ALDH1A1-positive cells (27.90% of total population; Gate 1) and among them a group with very high fluorescence level was noted (2.50% of total population; Gate 2). In contrast, the ALDH1A1-positive cells were not present in the parental W1 cell line (Figure 4A). Similarly, in the A2780PR1 cell line we observed subpopulation of ALDH1A1-positive cells (40.30% of total population; Gate 1) and among them a group of cells with very high fluorescence level was observed (2.90% of total population; Gate 2). The parental A2780 cell line were negative for ALDH1A1 (Figure 4B). The shift in the fluorescent signal in some cells indicates that there is not simply an overall increase of ALDH1A1 activity in the whole cell population but rather that subpopulations of ALDH1A1-medium positive, ALDH1-high positive, and ALDH1A1-negative cells are present. These results correlate with the fluorescence analysis observations.

### 2.5. Coexpression of P-gp and ALDH1A1

**Figure 5:**
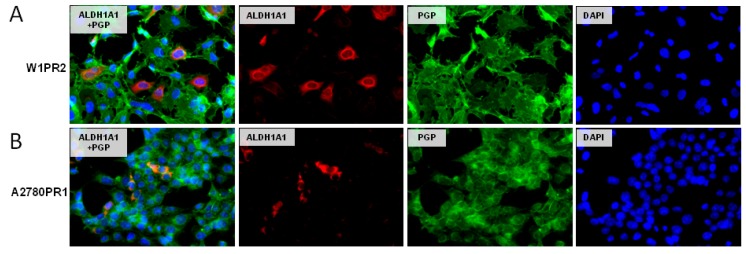
Immunofluorescence visualization of P-gp and ALDH1A1 coexpression in the W1PR2 (**A**) and A2780PR1 (**B**) cell lines. P-gp was detected using the anti-P-gp antibody and Alexa Fluor^®^488-conjugated secondary antibody (green). ALDH1A1 was detected using the anti-ALDH1A1 antibody and Alexa Fluor^®^594-conjugated secondary antibody (red). To visualize the cell nuclei, the cells were mounted with a DAPI-containing mounting medium (blue). Objective 40×.

One of the characteristic features of CSCs is expression of drug transporters from ABC family. Therefore, we investigated drug-resistant cell lines in order drug transporters expression. Previously we observed increased expression of BCRP in all cells from the W1TR cell line [49]. Here we showed that W1PR2 (Figure 5A) and A2780PR1 (Figure 5B) cell lines express P-gp at a high level and that the expression is uniform in the whole cell population. On the contrary, the ALDH1A1 expression was observed only in some cells (Figure 5A,B). This observation is in accordance with CSCs model of drug resistance development.

### 2.6. Coexpression of LOX and Collagens

**Figure 6:**
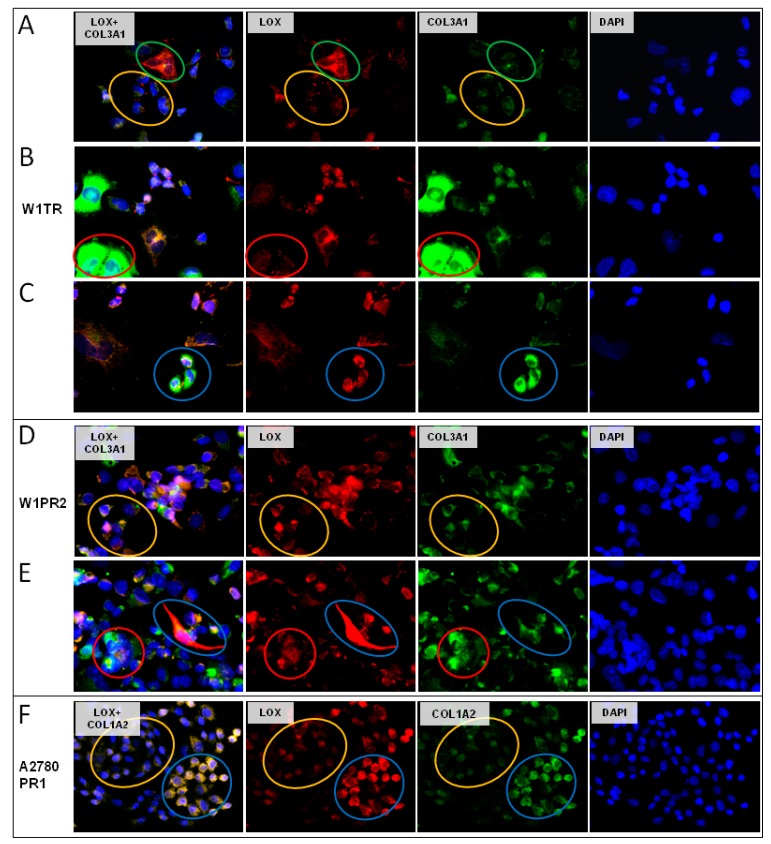
Immunofluorescence visualization of LOX and COL3A1 coexpression: (**A**) W1TR—low LOX/low COL3A1, yellow circle and high LOX/low COL3A1, green circle; (**B**) W1TR—low LOX/high COL3A1, red circle; (**C**) W1TR—high LOX/high COL3A1, blue circle; (**D**) W1PR2—low LOX/low COL3A1, yellow circle; (**E**) W1PR2—low LOX/high COL3A1, red circle; (**F**) A2780PR1—low LOX/low COL1A2, yellow circle and high LOX/high COL1A2, blue circle. LOX was detected using the anti-LOX antibody and Alexa Fluor^®^594-conjugated secondary antibody (red). COL3A1 was detected using anti-COL3A1 antibody and Alexa Fluor^®^488-conjugated secondary antibody (green). To visualize the cell nuclei, the cells were mounted with a DAPI-containing mounting medium (blue). Objective 40×.

Since LOX is involved in collagen metabolism [30], we investigated whether both proteins could be expressed in the same cells. As the result of the immunofluorescence coexpression experiments we could observe four different cell populations in W1TR cell line. Cells with low expression of LOX and low expression of COL3A1 (Figure 6A, yellow circle), cells with high expression of LOX and low expression of COL3A1 (Figure 6A, green circle), cells with high expression of COL3A1 but low expression of LOX (Figure 6B, red circle), as well as cells with high expression of both proteins (Figure 6C, blue circle). Similar cell populations were observed in W1PR2 cell line. We observed cells with low level of LOX and COL3A1 expression (Figure 6D, yellow circle), high level of COL3A1 but low level of LOX (Figure 6E, red circle), and cells expressing medium level of COL3A1 but very high level of LOX (Figure 6E, blue circle). In A2780PR1 cell lines we observed two cell populations. Cells with a low level of LOX expressed a lower level of COL1A2 (Figure 6F, yellow circle) and cells with high level of LOX expressed a higher level of COL1A2 (Figure 6F, blue circle).

### 2.7. Coexpression of ALDH1A1 and Collagens

**Figure 7:**
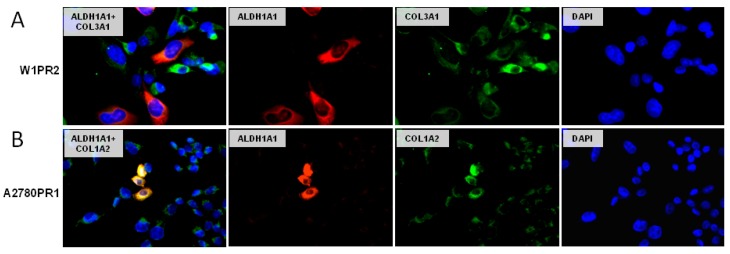
Immunofluorescence visualization of ALDH1A1 and COL3A1 coexpression in the W1PR2 cell line (**A**) and coexpression of ALDH1A1 and COL1A2 in the A2780PR1 cell line (**B**). ALDH1A1 was detected using the anti-ALDH1A1 antibody and Alexa Fluor^®^488-conjugated secondary antibody (green). COL3A1 and COL1A2 were detected using the anti-COL3A1 and anti-COL1A2 antibodies and Alexa Fluor^®^594-conjugated secondary antibody (red). To visualize the cell nuclei, the cells were mounted with a DAPI-containing mounting medium (blue). Objective 40×.

In this study, we investigated ALDH1A1 and collagen coexpression in PAC-resistant cells. In the W1PR2 cell line we could observe expression of both proteins. All cells were positive for COL3A1 and some of them showed higher expression than others. Positive expression for ALDH1A1 protein was presented only by small group of cells. However, different cells were positive for ALDH1A1 and different for high expression of COL3A1 (Figure 7A). On the other hand, a clear correlation between ALDH1A1 and COL1A2 was observed in A2780PR1 cell line (Figure 7B).

### 2.8. Coexpression of LOX and ALDH1A1

**Figure 8:**
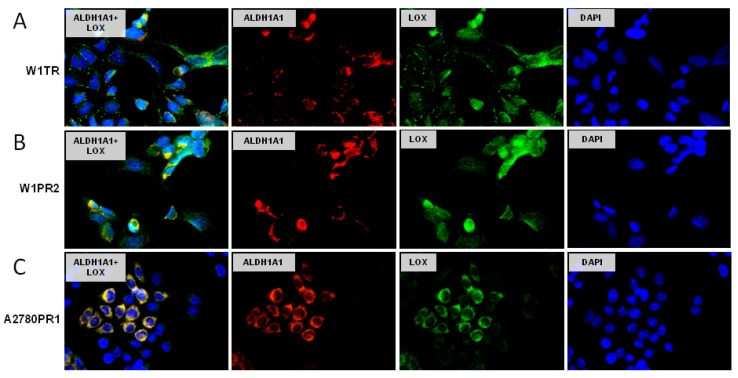
Immunofluorescence visualization of LOX and ALDH1A1 coexpression in the W1TR (**A**), W1PR2 (**B**), and A2780PR1 (**C**) cell lines. LOX was detected using the anti-LOX antibody and Alexa Fluor^®^488-conjugated secondary antibody (green). ALDH1A1 was detected using the anti-ALDH1A1 antibody and Alexa Fluor^®^594-conjugated secondary antibody (red). To visualize the cell nuclei, the cells were mounted with a DAPI-containing mounting medium (blue). Objective 40×.

We were also interested whether the same cells can express LOX and ALDH1A1 simultaneously. The double immunofluorescence assay showed the coexpression of LOX and ALDH1A1 in W1TR cell line. The ALDH1A1 positive cells expressed much higher levels of LOX than the cells without ALDH1A1 expression (Figure 8A). A similar correlation was also observed in the W1PR2 cell line (Figure 8B). However, the highest correlation between LOX and ALDH1A1 expression was observed in A2780PR1 cell line (Figure 8C).

### 2.9. Immunohistochemical Expression of LOX, COL1A2, COL3A1, COL21A1, and ALDH1A1 Proteins

**Figure 9:**
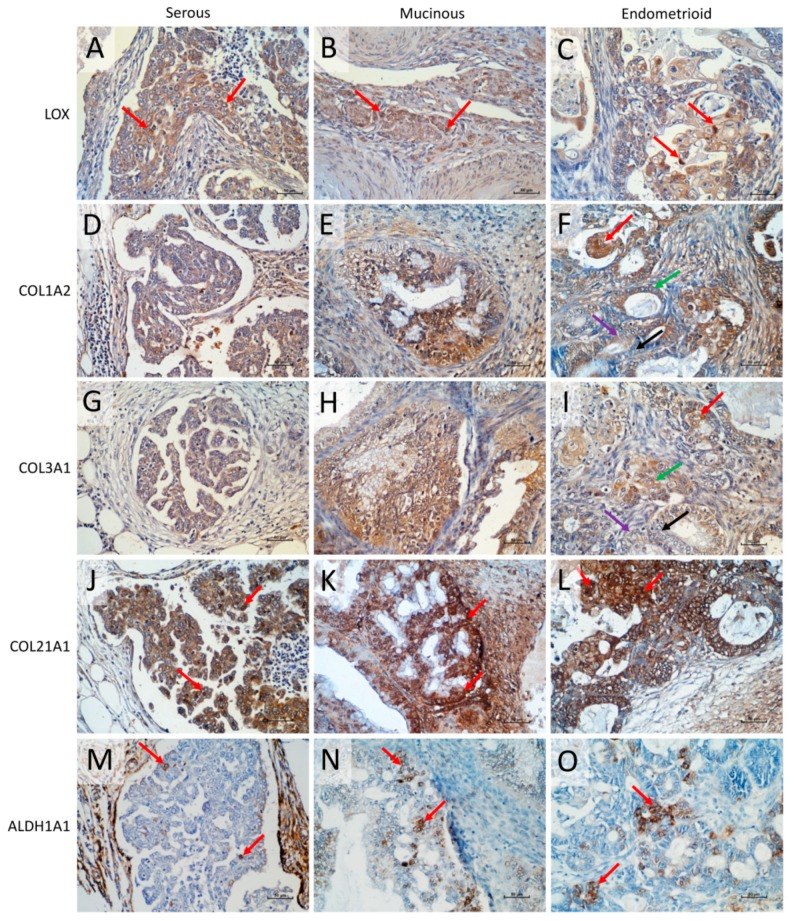
Immunohistochemical expression of (1) LOX in serous adenocarcinoma (**A**), mucinous ovarian cancer (**B**), and endometrioid adenocarcinoma (**C**), strong cellular LOX expression illustrated by red arrows; (2) COL1A2 in cancer cells of serous adenocarcinoma (moderate intensity score) (**D**), mucinous ovarian cancer (strong intensity score) (**E**), and endometrioid adenocarcinoma: cells with high intensity score (red arrow), cells with moderate intensity score (green arrow), cells with mild intensity score (purple arrow), and cells with no COL1A2 expression (black arrow) (**F**); (3) COL3A1 in cancer cells of serous adenocarcinoma (moderate intensity score) (**G**), mucinous ovarian cancer (strong intensity score), (**H**) and endometrioid adenocarcinoma: cells with high intensity score (red arrow), cells with moderate intensity score (green arrow), cells with mild intensity score (purple arrow), and cells with no COL1A2 expression (black arrow) (**I**); (4) COL21A1 in cancer cells of serous (**J**), mucinous (**K**), and endometrioid (**L**) carcinoma: cells with stronger expression illustrated by red arrows; (5) ALDH1A1 in serous adenocarcinoma (**M**), mucinous ovarian cancer (**N**) and endometrioid adenocarcinoma (**O**), strong cellular ALDH1A1 expression illustrated by red arrows. Sections were counterstained with hematoxylin. Scale bar = 50 μm.

The immunohistochemical analysis of LOX, COL1A2, COL3A1, COL21A1, and ALDH1A1 was performed for few cases of endometrioid, serous, and mucinous ovarian cancer in order to verify the localization of analyzed proteins in the real cancer tissue. The results obtained for LOX immunohistochemistry indicated the stronger expression of the protein in cancer cells than in extracellular matrix (Figure 9). Furthermore, among the tumor cells we could observe cells with higher LOX expression level (moderate/strong IRS score) when compared to other cancer cells (mild/moderate IRS score) regardless the carcinoma type (Figure 9A–C, red arrows).

The immunohistochemical analysis of COL1A2, COL3A1, and COL21A1 revealed that regardless of the type of analyzed ovarian cancer subtype the expression of those proteins was present not only in the ECM surrounding the tumor (what was expected) but also in cancer cells. And what is more, the expression of all collagen proteins was stronger in cancer cells than in ECM. For the COL1A2 all ovarian cancer subtypes expressed the protein, with moderate expression in serous carcinoma (Figure 9D) and strong in mucinous and endometriod carcinoma (Figure 9 E,F). However, in the endometrioid carcinoma, we could observe the subpopulations of cells presenting different intensity score from cells with a high intensity score (Figure 9F, red arrow), through cells with moderate (Figure 9F, green arrow) and mild (Figure 9F, purple arrow) intensity scores, up to cells with no COL1A2 expression (Figure 9F, black arrow). Similar IHC result was observed for COL3A1 expression. We noted moderate expression in serous carcinoma (Figure 9G) and moderate to strong in mucinous and endometrioid carcinomas (Figure 9H,I). Likewise, the endometrioid carcinoma revealed subpopulations of cells with high intensity score (Figure 9I, red arrow), moderate (Figure 9I, green arrow), and mild (Figure 9I, purple arrow) intensity scores, and finally cells with no COL3A1 expression (Figure 9I, black arrow). The last type of analyzed collagen type was COL21A1. The protein was present in cancer cells of all types of examined carcinomas (Figure 9J–L). The expression of COL21A1 was the strongest among all investigated collagens. However, some cells presented stronger COL21A1 expression then other ones in all types of carcinomas (Figure 9J–L, red arrows). The correlation analysis of collagens expression with tumor stage requires wider analysis and will be proceed in the next step of our experiments.

We have also performed immunohistochemical analysis for ALDH1A1 protein. Regardless of the type of ovarian carcinoma we could observe the population of cells with strong immunohistochemical signal (Figure 9M–O).

## 3. Discussion

Late diagnosis and development of drug resistance are the main reasons of treatment failure in epithelial ovarian cancer (EOC) [2,4,5,6]. Development of drug resistance takes place at both, single cell as well as cancer tissue level. The CSC-model explained the development of drug resistance at the cellular level based on the self-protecting mechanisms including high expression of drug transporters [44,45] and high level of ALDH1A1 [43,45,46]. The ECM model of drug resistance development postulates that small populations of tumor cells express ECM molecules at high levels and only these cells survive chemotherapy because of CAM-DR [21,22,23,24,25,26,27,50]. The presence of CSCs [47,48,49,51,52] and expression of ECM molecules by cancer cells [23,24,25,26] has been also observed in drug-resistant cancer cell lines. However, these observations were made separately. Recently, for the first time we have noted increased expression of ECM molecule (COL3A1) in ALDH1A1 positive cells [27].

On the basis of the previous microarray analysis results we have selected another ECM molecule with elevated expression in drug-resistant cell lines [26]. The current study revealed a statistically significant increase in *LOX* mRNA level in the three drug-resistant cell lines (PAC- and TOP-resistant) in comparison to their parental drug-sensitive cell lines. The big differences in expression level observed among investigated cell lines were confirmed at the protein level. The fluorescence analysis showed that drug-resistant A2780PR1, W1TR, and W1PR2 cell lines were not homogenous in regard to LOX protein expression and two cell populations with medium and very high LOX expression were observed. The obtained results directed us to conduct a careful literature search in order elucidate a relation of LOX expression with drug resistance development. Unfortunately, we could not find any research that compares levels of LOX expression between drug-sensitive and resistant pairs of cell lines. Therefore, we could not refer our results to others.

However, LOX overexpression was reported in different types of cancer cell lines, e.g., colorectal cancer [53], primary clear cell renal cell carcinoma [54], esophageal squamous cell carcinoma [55], and ovarian cancer cell lines-OVCAR3 [56] and SKOV-3 [57]. In all instances elevated levels of LOX led to increased proliferation, migration, and invasion of cancer cells. With regard to the role of LOX in drug resistance, it has been reported that knockdown of LOX gene resulted in increased sensitivity to microtubule targeting cytotoxic drugs: PAC, docetaxel, and vincristine [58]. In a few recent papers, we have shown the increased expression of genes in response to short time PAC [59,60], TOP [60,61,62], and CIS [62] treatment in drug-sensitive ovarian cancer cell lines. In this study W1 cell line showed clear time-dependent increase in *LOX* mRNA level after short time PAC treatment. The research of time and dose dependent influence of cytotoxic drugs on resistance development are very rare but turned out very significant. From our experience genes overexpressed at the very beginning of cell exposure to the drug remain at a very high level in drug-resistant cell lines pointing their putative role in drug resistance mechanism.

As mentioned earlier the development of drug resistance in CSCs model is associated with the expression of ALDH1A1 by cancer stem cells [43,63]. The results of our studies revealed increase in ALDH1A1 expression in TOP- and PAC-resistant cell lines. However, the fluorescence analysis demonstrated that cellular ALDH1A1 expression is not uniform for considered cell line; however, two subpopulations could be distinguished. All three investigated cell lines were characterized by subpopulation of highly positive and weak to negative cells. In contrast, both parental drug-sensitive cell lines were completely negative. This result was confirmed by quantitative Aldeofluor assay, where only ~2–3% of W1PR2 and A2780PR1 cells showed strong ALDH+ fluorescent signal and ~30–40% medium fluorescence intensity and ~4–5% for W1TR, as described previously [49]. A similar observation was made by others, where the presence of ALDH1A1 positive cells was demonstrated in A2780 CIS-resistant and SKOV-3 taxane-resistant ovarian cancer cell lines [51]. Also, ALDH+ cells isolated from tumor of ovarian cancer patients were more resistant to chemotherapy then ALDH- cells [48,64]. The immunohistochemical analysis of ovarian cancer tissues in this experiment also revealed the subpopulation of cells with noticeable strong ALDH1A1 signal found among surrounding ALDH1A1 negative cancer cells. One of the characteristic features of CSCs in the context of drug resistance is expression of drug transporters [45]. In our study, we have noticed that development of drug resistance was associated with BCRP expression in TOP resistant cell line [16,49] and P-gp expression in PAC resistant cell lines as we showed here and previously [12,13]. Although all the investigated cells express BCRP [49] or P-gp it was observed that only a small population showed coexpression of drug transporters and ALDH1A1 simultaneously. Since, we have demonstrated that investigated cell lines present features of CSCs model of drug resistance, in the second part of our study we have focused on molecules related to ECM model. As mentioned earlier, all drug-resistant cell lines showed differential levels of LOX expression. Previously we could observe the same cell lines presenting differential expression of collagen genes and proteins (e.g., COL3A1, COL1A2, and COL21A1) [27,65]. Hereby, we have combined both experiments and showed that among investigated drug-resistant cell lines different subpopulations of cells expressing simultaneously LOX and collagens could be found. TOP- and PAC-resistant cell lines derived from W1 cell line presented variable COL3A1 and LOX coexpression whilst A2780 derived PAC-resistant cell line showed clear correlation between COL1A2 and LOX expression. It remains interesting why homogenous cell lines present subpopulations of cells with different COL3A1 or COL1A2 and LOX expression. The diverse gene expression can result in different functions of those, theoretically identical, cells and points that cancer cells growing in monolayer reflect some features of cancer tissue in response to cytotoxic conditions.

The expression of LOX and collagens as the reaction of cells to TOP and PAC treatment suggests their role in acquiring resistance to those drugs. That remains in accordance with Morin et al. pointing the role of ECM molecules in drug resistance development [22]. The possible roles of collagens in drug resistance can be related to direct drug binding [20], physical drug diffusion blocking [66,67], and induction of CAM-DR [21,22] and were discussed in more details previously [27,65]. The expression of LOX in drug-resistant cell lines seems to be related to high collagen expression as in physiological condition LOX is involved in collagen cross-linking increasing ECM stiffness [30]. Previously we observed that COL3A1, COL1A2 and COL15A1 were present in cell culture media of drug-resistant cell lines [65,68]. Furthermore, in TOP-resistant cell line COL3A1 was secreted to cell culture medium forming structure similar to spider’s web [27] that could be a step ahead of collagen cross-linking. This result is supported by Di Stefano et al. since they proved that cell culture media from LOX expressing cells significantly increase the collagen matrix stiffness [55]. The in vivo experiments also prove a role of LOX in collagen cross-linking and next in drug resistance enhancement [20,66,67,69]. Therefore, it can be stated the more stiffen collagen network the more efficient blocking of drug diffusion [20,66] and/or more efficient CAM-DR induction.

**Figure 10:**
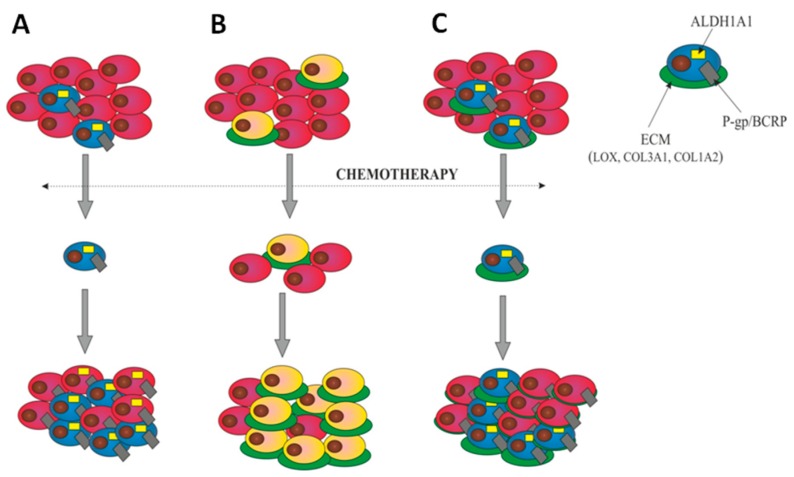
Models of drug resistance development. (**A**) Cancer stem cells (CSCs) model—a small population of cells, characterized by ALDH1A1 expression (blue) is intrinsically resistant to chemotherapy. These cells present high levels of ABC transporters like P-gp and/or BCRP and other drug-resistant proteins. Only these cells survive chemotherapy. After therapy, they divide and repopulate the tumor mass composed of the stem cells and differentiated cells derived from the stem cells and resistant to chemotherapy as well. The number of CSCs after chemotherapy increases. (**B**) The ECM-related model suggests that cells with a high level of ECM component expression (yellow/green) are resistant to chemotherapy. Only the cells with high ECM expression survive therapy and are able to induce resistance in neighboring cells via CAM-DR. The content of cells expressing ECM molecules after chemotherapy increases. (**C**) Our model of CSCs/ECM establishes that ALDH1A1-positive CSCs express a high level of drug transporters like P-gp or BCRP as well as high level of ECM proteins (blue/green). These cells survive chemotherapy and then divide and repopulate the tumor mass. After therapy the content of CSCs/ECM cells in the tumor mass increases and all of them are resistant because of the high level of drug transporters expression. Most of CSCs demonstrates the high level of ECM molecules protecting them, and surrounding cells (CAM-DR), against chemotherapy.

The final step in our study was to determine whether the investigated drug-resistant cell lines, presenting features of CSCs and ECM-related models of drug resistance separately, could present these models simultaneously. We have proved the clear coexpression of ALDH1A1 and some types of collagens in cell lines resistant to TOP (COL3A1 coexpression in W1TR) and PAC (COL1A2 coexpression in A2780PR1) [27]. Even more unequivocal result was obtained for simultaneous expression of ALDH1A1 and LOX, where high level of LOX was present only in ALDH1A1 positive cells in all examined drug-resistant cell lines. Thus, the ALDH1A1 positive cells seems to play a crucial role in ECM molecules production in our model. According the CSCs model, the ALDH1A1 positive cells are responsible for drug resistance development and tumor progression [43,44,45,46]. On the other hand, the ECM model establishes the role of ECM-producing cells as responsible for development of drug resistance and its induction in surrounding cells via CAM-DR mechanism [21,22]. Therefore, in this paper we have described two distinct models of drug-resistant CSCs and ECM-observed by us and others independently (Figure 10). However, no one has previously established the hypothesis that those models can occur simultaneously. The results demonstrated in this paper indicates the LOX and collagens as important factors that might contribute to cancer resistance mechanism. The increased expression of ECM genes/proteins by ALDH1A1 positive cells gives evidence that cancer cells exposed to paclitaxel and topotecan are able to develop a new mechanism of drug resistance combining the models known so far as separate ones (CSCs and ECM). Since the results presented in this paper are based on the basic research this hypothesis needs more extensive investigation and confirmation by the functional analysis. Therefore, as a continuation of current research a knockdown and overexpression experiments with the use of animal model of ovarian cancer will be proceed.

As an additional step in our study we have analyzed the examined proteins in ovarian cancer tissues. LOX and collagens are considered as extracellular proteins where they localize and are involved in cell adhesion and ECM remodeling. Since their extracellular presence is not unusual it remains interesting that cancer cells of solid tumors also revealed expression of those proteins, and an even stronger signal was observed for LOX in cancer cells than in extracellular matrix. A similar observation was also made by others where in patients with early lung adenocarcinoma LOX tumor cell expression was considered as an independent biomarker of poor prognosis whereas stromal staining was not associated with invasive morphology or survival [36]. LOX expression was also associated with progression, invasion, and drug resistance in breast [37,38], colorectal [54], and ovarian cancer patients [40,57,70]. The same ovarian cancer specimens were examined in regard to collagens expression. Regardless of the type of ovarian cancer subtype the expression of proteins was present not only in the ECM surrounding the tumor but also—and even stronger—in cancer cells. Among the investigated collagens the expression of COL1A2 was only previously noted in gastric cancer where was associated with metastasis formation and advanced stages of the disease [71]. The best of our knowledge this research for the first time describes the expression of COL1A2 in ovarian cancer cells of the tumor. However, the most abundantly expressed type of collagen in the investigated ovarian cancer samples was COL21A1, which is responsible for three-dimensional structure of dense connective tissue [72]. Moreover, this is also for the first time when expression of COL21A1 was noted not only in ovarian cancer but also in any other cancers. In contrast, the expression of COL3A1 was previously indicated in breast cancer where was associated with tumor development and progression [73] and with the resistance to CIS in ovarian cancer [74]. Furthermore, similarly to drug-resistant cell lines also in examined cancer tissue samples, the expression of investigated molecules was not uniform and differed between cancer cells in the same tumor. Therefore, we hypothesize that expression of LOX and collagens in ovarian cancer tissues may be associated with drug resistance since LOX is responsible for the collagen cross-linking increasing ECM stiffness [30].

## 4. Materials and Methods

### 4.1. Reagents and Antibodies

Culture media (RPMI-1640 and MEM), fetal bovine serum, antibiotic–antimycotic solution, l-glutamine, DAPI mounting medium and PAC and TOP were purchased from Sigma (Sigma-Aldrich, Poznań, Poland). Rabbit polyclonal anti-LOX Ab was obtained from Proteintech (Proteintech Europe, Manchester, UK), rabbit monoclonal anti-ALDH1A1 Ab was purchased from Abcam (Abcam, Cambridge, UK), mouse monoclonal anti-ALDH1A1, and goat polyclonal anti-COL1A2, -COL3A1, and -COL21A1 Abs were purchased from Santa Cruz Biotechnology (Santa Cruz Biotechnology Inc, Dallas, TX, USA). Mouse monoclonal anti-PGP Ab was purchased from Invitrogen (Thermo Fisher Scientific, Waltham, MA, USA). Donkey anti-goat horseradish peroxidase (HRP)-conjugated Ab was purchased from Santa Cruz Biotechnology (Santa Cruz Biotechnology Inc., Dallas, TX, USA). The fluorescent MFP488 donkey anti-goat IgG was obtained from MoBiTec (MoBiTec, Molecular Biotechnology, Goettingen, Germany, MFP488) and fluorescent Alexa Fluor^®^488 and Alexa Fluor^®^594 Donkey Anti-Rabbit IgG from Jackson ImmunoResearch Laboratories (Jackson ImmunoResearch Laboratories, Cambridgeshire, UK). Western blot reagents (membranes, gels and protein marker) were purchased from Bio-Rad (Bio-Rad Laboratories Ltd., Watford, Hertfirdshire, UK).

### 4.2. Cell Lines

Two ovarian cancer cell lines were used in this study. A2780 human epithelial ovarian cancer cell line was obtained from ATCC (American Type Culture Collection, Manassas, VA, USA) and W1 primary ovarian cancer cell line was established in our laboratory using ovarian cancer tissue obtained from an untreated patient. All drug-resistant derivatives of A2780 and W1 cells were developed by exposing them to cytotoxic drug treatment. PAC resistant sublines of A2780 (A2780PR1) and W1 (W1PR2) cell lines and TOP resistant W1 subline (W1TR) were developed by subjecting those cells to incremental doses of PAC or TOP. The final concentrations used for selecting the PAC resistant A2780 and W1 cell lines were 300 ng/mL and 1100 ng/mL of PAC, respectively. The final concentration of TOP for W1TR was 24 ng/mL of TOP. The increase in resistance in relation to parental drug-sensitive cell lines was as follows, 491-fold for A2780PR1 vs. A2780, 20-fold for W1TR vs. W1, and 967-fold for W1PR2 vs. W1 as described previously [13,75]. All cell lines were cultured in appropriate media (MEM for A278 and RPMI-1640 for W1), supplemented with 10% FBS, 2 pM l-glutamine, penicillin (100 units/mL), streptomycin (100 units/mL), and amphotericin B (25 μg/mL) at 37 °C in a 5% CO_2_ atmosphere.

### 4.3. QPCR Gene Expression Analysis

RT-qPCR on cultured cells was performed as standard in regard to analyze *LOX* and *ALDH1A1* gene expression. Total RNA was extracted from A2780, W1, and drug-resistant cell lines using the GeneMATRIX Universal RNA purification kit (EURx Ltd., Gdansk, Poland) and followed by reverse transcription using M-MLV reverse transcriptase (Invitrogen by Thermo Fisher Scientific, Waltham, MA, USA), as described by the manufacturer’s protocols. cDNA synthesis was performed on 2 μg of RNA and real-time PCR was performed using sequence-specific primers indicated in Table 1 and the 7900HT Fast Real-Time PCR System (Applied Biosystems, Foster City, CA, USA), Maxima SYBR Green/ROX qPCR Master Mix (Thermo Fisher Scientific, Waltham, MA, USA) according to procedure described previously [65]. Gene expressions were analysed using the relative quantification (RQ) method with drug-sensitive A2780 or W1 cell lines used as the calibrator [65]. After amplification, melting curves were used to determine the specificity of the gene products, which was confirmed by running the PCR products on 3% agarose gel.

### 4.4. Protein Isolation and Western Blot Analysis

Cells (1 × 10^6^ cells/25 μL lysis buffer) were lysed using RIPA buffer containing a protease inhibitor cocktail (Roche Diagnostics GmbH, Mannheim, Germany) for 60 min on ice at 4 °C. The lysates were centrifuged at 8000× *g* for 10 min at 4 °C, and protein concentrations were determined using the Bradford protein assay system (Bio-Rad Laboratories, Hemel Hempstead, UK). Wells were loaded with 30 μg protein resuspended in 4x loading buffer (Bio-Rad Laboratories, Hemel Hempstead, UK). After separation on a 4–20% mini-PROTEAN^®^ TGX™ precast gel using the SDS-PAGE technique, proteins were transferred to a nitrocellulose membrane, blocked with 5% milk in TBS/Tween (0.1 M Tris-HCl, 0.15 M NaCl, 0.1% Tween 20) and probed using primary antibodies against LOX at a 1:1000 and ALDH1A1 at a 1:500 dilution and followed by incubation with HRP-labeled secondary antibodies. Signals were developed using chemiluminescence detection system (ECL, Femto Super Signal Reagent) and Hyperfilm ECL (GE Healthcare, Buckinghamshire, UK). The protein loading was normalized by reblotting the membranes with rabbit anti-GADPH Ab (Santa Cruz Biotechnology), at a 1:1000 dilution and goat anti-rabbit HRP-conjugated Ab (Santa Cruz Biotechnology).

### 4.5. ALDEFLUOR Assay

To measure aldehyde dehydrogenase activity (ALDH1A1) the ALDEOFLUOR kit was used according to the manufacturer’s protocol (Stem Cell Technologies Germany GmbH, Köln, Germany). The gates were determined by diethylaminobenzaldehyde (DEAB) treatment, an ALDH inhibitor that served as negative control. Stained cells were analyzed under flow cytometer and ALDH1A1-positive population was defined as the cells with increased FITC fluorescence.

### 4.6. Immunofluorescence Analysis

The cells were seeded into 24-wells chamber glass slides and grown to a near-confluent state. Immunofluorescence analysis was performed according the standard procedure. Briefly, cells were washed two times with PBS, fixed and permeabilized with ice-cold acetone/methanol (1:1) for 10 min, rinsed with PBS and blocked in 3% BSA for 30 min at room temperature. After blocking, cells were incubated in primary antibody solution against LOX (rabbit polyclonal anti-LOX antibody, 1:100, Proteintech, Manchester, UK) and ALDH1A1 (rabbit monoclonal anti-ALDH1A1 antibody, 1:100, Abcam, Cambridge, UK) for 2 h at room temperature. Afterwards, cells were washed three times with PBS and incubated in Alexa Fluor^®^488 secondary antibody solution (Donkey Anti-Rabbit IgG, Jackson ImmunoResearch Laboratories, Cambridgeshire, UK) for 1 h at room temperature. Slides were washed with PBS and mounted in DAPI mounting medium. The expression analysis and pictures were taken under fluorescence microscope (Zeiss Axio-Imager.Z1).

### 4.7. Double Immunofluorescence Analysis

For double fluorescence staining, the fixation, blocking and washing steps were conducted as described for immunofluorescence procedure. After blocking with 3% BSA the cells were incubated (2h, room temperature) with the mixture of two primary antibodies in subsequent pairs: (1) PGP (1:50) + LOX (1:100); (2) LOX (1:100) + ALDH1A1 (1:100); (3) LOX (1:100) + COL3A1 (1:100) or COL1A2 (1:100) or COL21A1 (1:100); (4) ALDH1A1 (1:100) + COL3A1 (1:100) or COL1A2 (1:100) or COL21A1 (1:100) (description of all antibodies in chapter ‘Reagents and Antibodies’). Then cells were washed with PBS and incubated in the mixture of two respective green dye-labeled (MFP488, donkey anti-goat IgG, 1:200, MoBiTec or Alexa Fluor^®^488, donkey anti-rabbit IgG, 1:400 Jackson ImmunoResearch Laboratories) and red dye-labeled (Alexa Fluor^®^594, donkey anti-rabbit IgG, 1:400, Jackson ImmunoResearch Laboratories) secondary antibodies (1 h, room temperature). Afterwards, the procedure was followed the same way as for immunofluorescence protocol.

### 4.8. Immunohistochemistry

Immunohistochemical staining was conducted for formalin-fixed, paraffin embedded human ovarian carcinoma patients’ samples. The slides with 5 µm sections were dewaxed with xylene and gradually hydrated. Sections were blocked in normal goat serum for 30 min and incubated in 1:200 of anti-LOX, or 1:100 anti-COL3A1, or -COL1A2 or -COL21A, and 1:100 anti-ALDH1A1 primary antibodies overnight at 4 °C followed by incubation with EnVision Detection System (Dako REAL^™^EnVision^™^ Detection System peroxidase/DAB+, Rabbit/Mouse, Dako, Glostrup, Denmark) or ImmPRESS^TM^ HRP Reagent Kit, peroxidase/DAB, anti-goat, Vector Lab, Burlingame, CA, USA) for 30 min at room temperature. Finally, slides were rinsed with distilled water, counterstained with haematoxylin, dehydrated, and mounted. Analysis of immunohistochemical staining was taken under the optical Olympus BH-2 microscope coupled to a digital camera. The expression of LOX and collagens was established in a way described previously [59] by mean proportion of immunopositive cancer cells and tumour surrounding tissue. The immunoreactivity score (IRS) was counted for 10 microscope fields for each specimen and derived by multiplying intensity score and distribution score.

### 4.9. PAC and TOP Response in Time Course Experiment

The time course experiments where drug-sensitive A2780 and W1 cells were subjected to PAC and TOP treatment for a short time were conducted as described previously [59]. Shortly, cells were seeded into 6-well plates at 0.5 × 10^6^ in 1ml of medium per well and treated with low concentrations of PAC (20 ng/mL and 25 ng/mL) or TOP (10 ng/mL and 20 ng/mL), respectively. The experiment was proceeded for 24, 48, and 72 h and after each period cells were harvested and the RNA was isolated.

### 4.10. Statistical Analysis

All data obtained in the experiments were analyzed using Student’s *t*-test. The statistical significance interval was determined at *p* ˂ 0.05.

## 5. Conclusions

In this report we have studied the expression of LOX, collagens, and ALDH1A1 in drug-resistant ovarian cancer cell lines. LOX and collagens belong to ECM molecules that can contribute to ECM related drug resistance, and on the other hand ALDH1A1 is a marker of CSCs model of drug resistance. It is remarkable that in our study those molecules were found to be coordinately expressed by cells resistant to PAC (LOX, ALDH1A1, and COL1A2) or TOP (LOX, ALDH1A1, and COL3A1). This finding suggests that different—and until now considered as independent—pathways of drug resistance development can be activated simultaneously in ovarian cancer cells. This represents the study where molecules related with CSCs (ALDH1A1) and ECM (LOX, collagens) models of drug resistance are described as occurring simultaneously in ovarian cancer cells treated with PAC and TOP.

## Figures and Tables

**Table 1:** Oligonucleotide sequences used for real-time quantitative RT-PCR (RQ-PCR) analysis.

Transcript	Sequence (5′-3′ Direction)	ENST Number http://www.ensembl.org	Product Size (bp)
LOX	CAGAGGAGAGTGGCTGAAGG CCAGGACTCAATCCCTGTGT	00000231004	116 bp
ALDH1A1	GTTGTCAAACCAGCAGAGCA CTGTAGGCCCATAACCAGGA	00000165092	115 bp
GADPH	GAAGGTGAAGGTCGGAGTCA GACAAGCTTCCCGTTCTCAG	00000229239	199 bp
β-actin	TCTGGCACCACACCTTCTAC GATAGCACAGCCTGGATAGC	00000331789	169 bp
HRPT1	CTGAGGATTTGGAAAGGGTG AATCCAGCAGGTCAGCAAAG	00000298556	156 bp
β2M	CGCTACTCTCTCTTTCTGGC ATGTCGGATGGATGAAACCC	00000558401	133 bp

## Abbreviations

LOX	Lysyl Oxidase
ALDH1A1	Aldehyde Dehydrogenase 1
ECM	Extracellular Matrix
CSCs	Cancer Stem Cells
CAM-DR	Cell Adhesion Mediated Drug Resistance
TR	Topotecan Resistance
PR	Paclitaxel Resistance
